# Enteric Neurons and Glial Cells Are Partially Protected in P2X7 Receptor‐Deficient Mice With Ulcerative Colitis

**DOI:** 10.1111/nmo.70499

**Published:** 2026-11-10

**Authors:** Roberta Figueiroa Souza, Marcos Antônio Ferreira Caetano, Caroline Bures De Paulo, Patricia Castelucci

**Affiliations:** ^1^ Department of Anatomy, Institute of Biomedical Sciences University of São Paulo São Paulo Brazil; ^2^ Department of Surgery, School of Veterinary Medicine and Animal Sciences University of São Paulo São Paulo Brazil

**Keywords:** enteric nervous system, inflammasome, inflammatory bowel diseases, neurogastroenterology, purinergic signaling

## Abstract

**Background:**

Inflammatory bowel diseases, such as colitis, cause enteric nervous system dysfunction. Purinergic signaling through the P2X7 receptor (P2X7R) is activated during intestinal inflammation by extracellular ATP, but its role in chronic colitis remains unclear.

**Aim:**

Investigate the effects of an experimental colitis model on the ENS from mice knockout (KO) of P2X7R.

**Methods:**

Colitis was induced in wild‐type (WT) and KO mice by three cycles of Dextran Sodium Sulfate (DSS) (Cycles 1 and 2, 2% and Cycle 3, 1.5%) dissolved in drinking water for 5 days, followed by drinking water for the next 14 days (KO/DSS and WT/DSS groups). The KO/SHAM and WT/SHAM groups received water during the same period. The animals were euthanized after 57 days, and the distal colon was collected. Disease activity index and histological analysis were performed. Whole‐mount preparations of the myenteric plexus were analyzed by immunofluorescence for neuronal nitric oxide synthase (nNOS), acetylcholine transferase (ChAT), β3‐Tubulin, P2X7R, glial fibrillary acidic protein (GFAP), and S100β, and the inflammasome component NLRP3. Neuronal and glial densities were quantified.

**Key Results:**

Chronic DSS‐induced colitis decreased the nitrergic and cholinergic neurons, β3‐tubulin–ir neurons, and GFAP‐ir and S100β glial cells in WT/DSS and KO/DSS groups. P2X7R‐ir cells decreased in WT/DSS animals, and NLRP3‐ir cells increased mostly in WT/DSS, indicating inflammasome activation. There were changes in neuronal profile areas. These findings were significantly attenuated in P2X7R KO mice, indicating neuronal protection during chronic inflammation.

**Conclusions and Inferences:**

Experimental colitis affected the ENS of the distal colon. A slight neuronal attenuation of nNOS‐expressing neurons was observed in the P2X7RKO.

## Key Points

Inflammatory bowel diseases, such as colitis, cause loss of the enteric neurons. The binding of ATP to P2X7R opens the channel that leads to membrane depolarization. The objective of this study was to investigate the effects of chronic experimental colitis on enteric neurons and enteric glia in P2X7R KO mice. The results demonstrate that the DAI was slightly better in the KO group. nNOS‐expressing neurons were somewhat more protected compared to other neuronal classes. This study provides an original demonstration of the presence of the NLRP3 inflammasome in enteric neurons, with an observed increase in both the WT/DSS and KO/DSS groups.

## Graphical abstract

P2X7R knockout mice efficacy. Chronic DSS‐induced colitis decreased the nitrergic and cholinergic neurons, β3‐tubulin–ir neurons, and GFAP‐ir and S100β glial cells in WT/DSS and KO/DSS groups. P2X7R‐ir cells decreased in WT/DSS animals, and NLRP3‐ir cells increased mostly in WT/DSS, indicating inflammasome activation. These findings demonstrate that, in the KO/DSS group, the neuronal loss and DAI scores were less pronounced compared to the WT/DSS group, indicating relative protection.

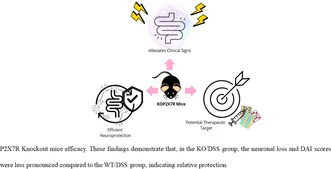

## 1 Introduction

The enteric nervous system (ENS) is characterized by the presence of neurons and glia organized into ganglia, arranged in two or more enteric plexuses, and interconnected neural pathways [1, 2]. The components of the ENS form integrated circuits that, either independently or together with extrinsic parasympathetic and sympathetic innervation, regulate various intestinal processes such as motility, transmucosal fluid movement, immune responses, and local blood flow [3, 4].

Enteric neuropathies can result from the loss, degeneration, and/or functional impairment of enteric neurons. They can be caused by congenital defects in the development of the ENS, acquired by infectious agents or toxins, or secondary to other pathological conditions, such as diabetes and neurodegenerative disorders [5, 6]. To study the chemical coding of myenteric plexus neurons, the enzyme neuronal nitric oxide synthase (nNOS) is used for inhibitory motor neurons; Calbindin (Calb) and CGRP for IPANs; and for cholinergic motor neurons, the enzyme choline acetyltransferase (ChAT) and/or Calretinin (Carl). For pan‐neuronal neurons, the marker anti‐HUC/D, PGP9.5, and/or β‐Tubulin are used [1, 3, 7].

Experimental colitis is a chronic idiopathic inflammatory bowel disease (IBD) of the colon that causes continuous superficial mucosal inflammation, extending from the rectum to the proximal colon [8, 9]. Although its etiology remains largely unknown, recent studies have observed that chronic inflammation may lead to epithelial cell stimulation and infiltration of immune cells and soluble mediators [8, 10, 11, 12, 13, 14].

When activated by bacterial antigens from the intestinal lumen, immune cells produce large amounts of pro‐inflammatory cytokines, such as tumor necrosis factor alpha (TNFα) and interleukin 1 beta (IL‐1β). They also release substantial bioactive molecules such as adenosine triphosphate (ATP), which further induce immune responses, resulting in inflammation and tissue damage in the colon [10, 15].

P2 receptors are classified into two subfamilies: P2X receptors, ligand‐gated ion channels, and P2Y, G‐protein‐coupled receptors [16, 17]. Among the seven P2X receptors (P2X1–7), the P2X7R is of particular interest in inflammation, with evidence showing its role in the inflammatory process [16, 18]. Nucleotides, including ATP and its receptors, have been implicated in the pathogenesis of IBD [17]. The binding of ATP to P2X7R opens the channel that leads to membrane depolarization, potassium (K^+^) efflux, and calcium (Ca^2+^) and sodium (Na^+^) influx [19]. These results support the idea that the P2X7R participates in the initiation and regulation of the inflammatory response. This occurs through the release of pro‐inflammatory cytokines, such as IL‐1β and TNFα [19, 20, 21], especially under conditions of inflammatory stress and cellular damage attributed to acute or chronic intestinal inflammation. In these cases, increased extracellular ATP levels lead to P2X7R activation [16, 22]. Also, ATP released by enteric glial cells during the inflammatory process acts on P2X7 receptors present in enteric neurons, promoting neuronal death and neuroinflammatory processes [23, 24, 25, 26].

Recent studies using knockout (KO) mice for the P2X7R gene suggest that this receptor is a viable therapeutic target for inflammatory diseases [26, 27, 28, 29]. P2X7R KO animals, when subjected to experimental ulcerative colitis, exhibited improvements in their general condition (without weight loss, colon shortening, or clear tissue damage), in addition to presenting protective effects on the intestinal barrier, without a decrease in goblet cells [28, 29, 30].

High concentrations of ATP open large pores that release inflammatory mediators, such as IL‐1β and TNFα. These cytokines are the main players in mediating inflammatory responses in colitis, regulated by the nuclear transcription factor kappa B (NF‐kB) pathway and the oligomerization of the leucine‐rich repeat nucleotide‐binding domain and pyrin domain‐containing protein 3 (NLRP3), the inflammasome multiprotein complex [10, 31, 32].

Inflammasomes are protein complexes important in several inflammatory processes [33]. Depending on the protein scaffold, 10 different types of inflammasome are known. The NLRP3 inflammasome is the most commonly activated in immune cells in response to pathogen‐associated molecular patterns (PAMPs) or damage‐associated nuclear patterns (DAMPs) [34].

Extracellular ATP induces IL‐1β production via NLRP3 by activating P2X7R, contributing to the pathogenesis of chronic inflammatory diseases and making it a key cytokine [35, 36].

Interest in P2X7R related to inflammatory diseases has been steadily growing in the literature. Given the previous studies on P2X7R [23] and the effects of its activation in the ENS, more specifically in enteric neurons and glial cells, it is interesting to see further studies elucidating the mechanisms of action involved in its activation.

Although studies have investigated DSS‐induced chronic colitis, the literature lacks research on the effects of this protocol on the various classes of enteric neurons, enteric glia, and the inflammasome in P2X7 receptor‐deficient mice. Thus, the rationale for this project is to explore the signaling mechanisms of P2X7R activation in the regulation of inflammatory responses in enteric neurons and enteric glial cells of the myenteric plexus, using P2X7R knockout mice subjected to chronic experimental ulcerative colitis.

## 2 Materials and Methods

### 2.1 Animals

This study was approved by the Ethics Committee on the Use of Animals (CEUA) of the Institute of Biomedical Sciences of the University of São Paulo (ICB/USP) under CEUA number 5491270323. A total of 74 animals were used in this study. C57BL/6 mice wild‐type (WT) and P2X7RKO deficient in the P2X7R gene (P2X7R−/−, P2X7RKO; B6.129P2‐R2rx7tm1Gab), male *Mus musculus* mice, were used. These mice were obtained from the Animal Facility of the Anatomy Department of the Institute of Biomedical Sciences (ICB) and the Animal Facility of the School of Medicine of the University of São Paulo. The animals were housed in the animal facility of the Department of Anatomy at the ICB at USP, aged 8–10 weeks, in a 24–12 h light/dark cycle, receiving water and food ad libitum. The animals were randomly allocated into four groups (*n* = 5): WT/SHAM Group, WT/DSS Group, KO/SHAM Group, and KO/DSS Group. Mice were euthanized with anesthetic overdose with a mixture of xylazine (20 mg/kg) and ketamine hydrochloride (100 mg/kg) administered subcutaneously. Female mice were not used in the study because the protocol involved chronic colitis, and the estrous cycle could have introduced variability into the results.

### 2.2 Experimental Colitis

Colitis was induced by three cycles [37, 38, 39] of 2% (2 g/100 mL) Dextran Sodium Sulfate (DSS) dissolved in water for 5 days (Cycles 1 and 2, 2% [2 g/100 mL] and 1.5% [1.5 g/100 mL] in Cycle 3), followed by drinking water for the next 14 days (WT/DSS and KO/DSS groups). The use of 1.5% DSS in Cycle 3 was intended to maintain the survival of animals (Figure S1).

The animals' condition was monitored daily by tracking body weight and bleeding, and recorded in the Disease Activity Index (DAI) for all experimental groups [38]. The WT/SHAM and KO/SHAM groups received only water at all times.

### 2.3 Disease Activity Index (DAI)

The disease activity index refers to the sum of scores assigned to the animals in each group related to body weight loss, stool consistency, and rectal bleeding. Weight loss (%) was scored (< 1%) as 0; (1%–2%) 1; (2%–4%) 2; (4%–6%) 3; and 4 (> 6%). Stool consistency was scored as normal (well‐formed pellets) (0), soft (1), loose and semi‐formed stools (2), liquid stools (3), and diarrhea (4). Rectal bleeding was scored as no blood (0), blood traces (1), mild bleeding (2), obvious bleeding (3), and gross bleeding (4) [40] (Table S1).

### 2.4 Immunofluorescence

For immunohistochemistry, distal colon segments were opened along the mesenteric border and rinsed with PBS. The tissues were then placed with their mucosal side down on a balsa wood plate and fixed overnight at 4°C with 4% paraformaldehyde in sodium phosphate buffer 0.2 mol/L (pH 7.3). Following fixation, the tissues were washed three times for 10 min each in 100% dimethylsulfoxide (DMSO) to clear the tissue to improve antibody penetration, then in PBS for 10 min, and stored at 4°C in PBS containing sodium azide (0.1%). The mucosa and circular layers were removed to prepare whole mounts of the longitudinal muscle layer with the myenteric plexus. For immunolabeling, samples were preincubated with 10% normal horse serum in PBS containing 1.5% Triton X‐100 for 45 min at room temperature. A combination of two antibodies was used for double labeling, as listed in Table S2. After primary antibody incubation for 48 h, tissues were washed in PBS three times for 10 min each and incubated with specific secondary antibodies for 1 h at room temperature (listed in Table S2). After the final PBS washes, tissues were mounted on slides with buffered glycerol containing 0.5 mol/L sodium carbonate (pH 8.6). Double‐labeling images were acquired by confocal microscopy on a Zeiss confocal scanning laser system installed on an LSM 800 Carl Zeiss Axio Imager. Z‐stacks of immunoreactive cells were recorded in optical sections with a center‐to‐center spacing of 0.2 μm using Corel Photo Paint and Corel Draw software programs.

### 2.5 Qualitative Analyses

Immunofluorescence techniques were employed to assess colocalization. Each antigen was first observed and photographed using a specific filter, followed by the labeling of the second antigen through a second filter. Subsequently, photomicrographs of both antigens were merged to evaluate colocalization.

### 2.6 Quantitative Analyses

The density of neurons (neurons/ganglion) labeling for P2X7R‐, nNOS‐, ChAT‐, β‐Tubulin‐, NLRP3‐, and enteric glial cells GFAP‐ir neurons per ganglion and the neuronal profile area were measured at 40 × magnification. Cells positive for each antigen were counted in 50 ganglia per animal. The measurement of the neuronal profile areas (μm^2^) was determined for 100 randomly selected neurons in two whole‐mount preparations per animal for the nNOS, ChAT, and β‐Tubulin markers (*n* = 5 animals per group, for each marker), totaling 2000 neuronal profile areas analyzed. Analysis was conducted using a Nikon 80i microscope equipped with a camera and NIS Elements AR 3.1 software (Nikon), and measurements were made using Image‐Pro Plus 4.1.0.0 software.

### 2.7 Histological Analyses

Samples of the distal colon from the WT Sham (*n* = 5), WT DSS (*n* = 5), KO Sham (*n* = 5), and KO DSS (*n* = 5) groups were washed in PBS and then opened along the mesenteric border. These samples were placed on balsa wood and fixed in 4% formaldehyde at 4°C for 48 h. Then, they were sectioned into 5 μm pieces and stained with hematoxylin–eosin (H&E), Periodic Acid‐Schiff (PAS) for goblet cells, and Picrosirius for collagen fibers for qualitative analysis of the changes induced by experimental ulcerative colitis. A Nikon 80i microscope equipped with a camera and NIS‐Elements AR 3.1 software (Nikon) was used for the analysis. The number of goblet cells was made in 10 intestinal crypts in four semi‐sequential histological sections (10 μm of the interval between sections) per animal (*n* = 3 animals per group) through PAS staining [41, 42]. Histomorphometric measurements—such as muscular, submucosa, crypt depth and crypt width—were performed according to Casagrande et al. [43]. The histomorphometric analysis was performed on images captured with a digital camera (Nikon) coupled to an optical microscope (Nikon 80i). Measurements were performed using Image‐Pro Plus software (Media Cybernetics, MD, USA). Images were acquired with a 20× objective to measure the muscular tunic, width and depth of the crypt, and submucosa. Sixteen measurements of each parameter were taken at four sections of three mice per group.

### 2.8 Statistical Analyses

The data were analyzed using two‐way analysis of variance (ANOVA), followed by Tukey's test for post hoc analysis. Results are presented as mean ± SEM. A significance level of 5% (*p* < 0.05) was adopted for all statistical analyses. The data were processed using GraphPad 8 software (Prism, USA).

## 3 Results

### 3.1 Mortality and Survival

During the 57 days of experimentation, we observed mice for mortality related to the progression of experimental colitis. All groups were analyzed, and mortality was recorded when mice died unexpectedly due to complications of ulcerative colitis or when they were euthanized due to advanced morbidity. Figure S1 is a Kaplan–Meier estimator showing the survival curve of the 74 animals used so far, with the relevant results demonstrating that: 69.56% of the mice in the WT/DSS group survived until the end of the experiment, with the highest mortality rates occurring on the 9th (78.26%) and 29th (69.5%) days, which remained until the end of the experiment. On the other hand, the animals in the KO/DSS group had a higher survival rate (85%), which occurred on the 9th day and remained until the end of the experiment. Both WT/SHAM and KO/SHAM groups finished the experiment with a 100% survival rate.

### 3.2 Disease Activity Index

The results presented are from the WT/SHAM, WT/DSS, KO/SHAM, and KO/DSS groups. The animals were weighed on the day of DSS ingestion (Day 0) and monitored throughout the three cycles of DSS, totaling 57 days of experimentation. Weighing always occurred at the same time. From the 4th day of DSS ingestion, the animals in the WT/DSS and KO/DSS colitis groups began to show weight loss, with 3 points in the KO/DSS group, which showed a marked loss on the 7th day, marking 4 points. In the WT/DSS group, weight loss also began on the 4th day, marking 3 points; however, in the KO/DSS group, the weight loss was less compared to the WT/DSS group. Furthermore, it is possible to observe that weight loss was more pronounced in the WT/DSS group (*p* < 0.05) (Figure 1A). During the 14 days when the animals received only drinking water, their weight normalized. However, weight decreased again on the 4th day of DSS intake in successive cycles (Cycle 2: Day 23 and Cycle 3: Day 43).

**FIGURE 1:**
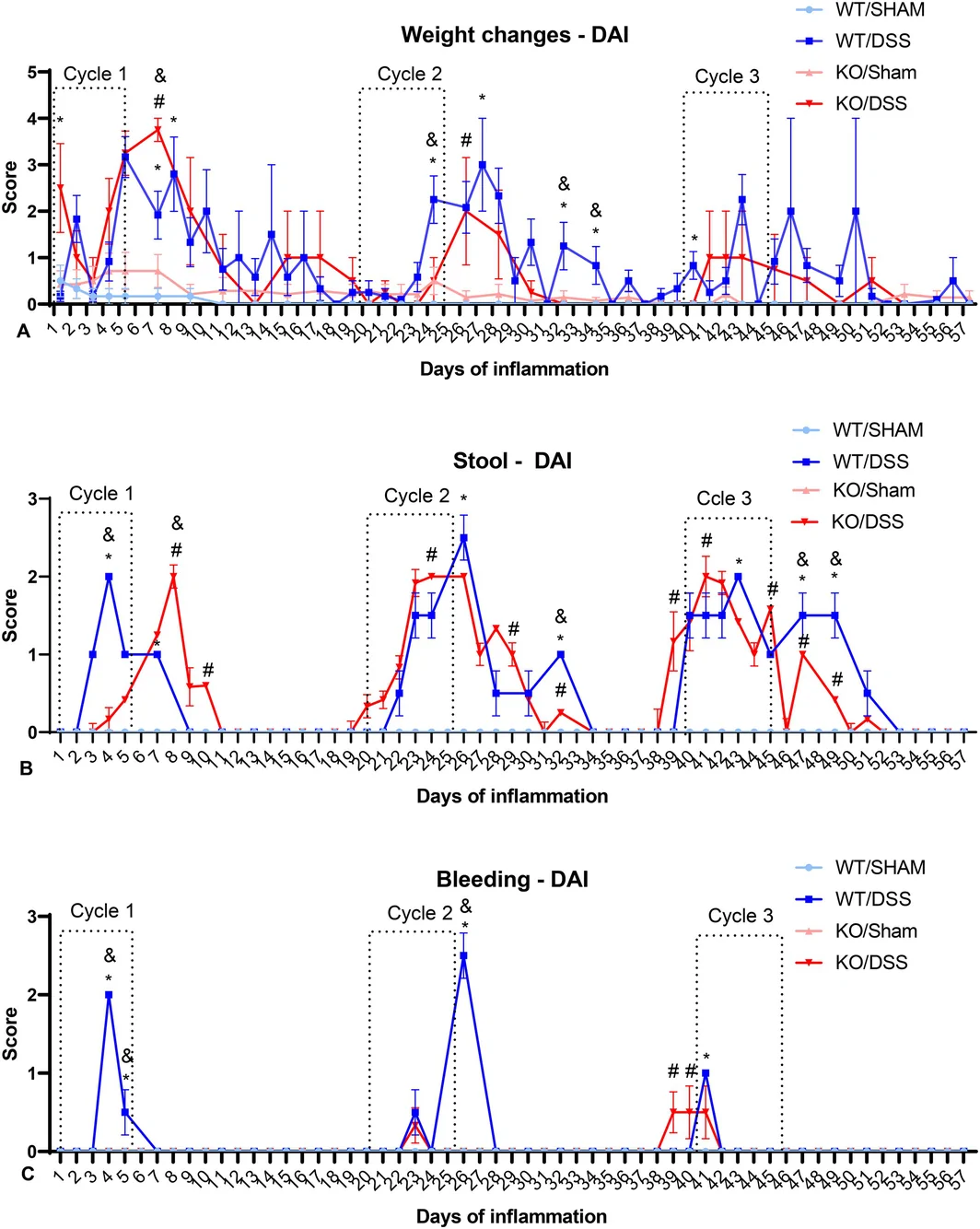
(A–C) DAIs of the animals in the WT/SHAM, WT/DSS, KO/SHAM, and KO/DSS groups. (A) Weight change, (B) stool consistency, (C) occult/gross bleeding. The data are presented as the means ± SEMs (*n* = 5 per group) and were compared by two‐way ANOVA and Tukey's multiple comparison test. *WT/SHAM versus WT/DSS (*p < 0.05), ^#^KO/Sham versus KO/DSS (^#^p < 0.05), ^&^WT/DSS versus KO/DSS (^&^ *p* < 0.05).

The KO/DSS animals lost weight compared to the KO/Sham group (Cycle 1: Day 7 and Cycle 2: Day 26, *p* < 0.05). There was a difference in weight between the WT/DSS and KO/DSS groups across the cycles (Cycle 1: Day 7, Cycle 2: Days 24, 32, and 34, *p* < 0.05) (Figure 1A).

Regarding stool consistency, the WT/SHAM and KO/SHAM groups presented well‐formed granules during the 57 days of experimentation. As for the colitis groups (WT/DSS and KO/DSS), there was a change in stool consistency starting on the 4th day in the WT/DSS group and starting on the 7th day in the KO/DSS group, where both groups scored 2 points (loose and semi‐formed stools). The highest score occurred on the 26th day (Cycle 2), where the animals in the WT/DSS group scored 3 points (loose stools) (*p* < 0.05) (Figure 1B).

There was a change in stool consistency in the 8th and 10th days (Cycle 1), 24th, 29th, and 32nd days (Cycle 2), 39th, 41st, 45th, 47th, and 49th days (Cycle 3) in the KO/DSS compared to KO/Sham (*p* < 0.05) (Figure 1B).

Also, there was a change in stool consistency in the 3rd and 8th day (Cycle 1), 24th and 32nd day (Cycle 2), 47th and 49th days (Cycle 3) in the WT/DSS compared to KO/DSS (*p* < 0.05) (Figure 1B).

Regarding bleeding, the highest scores were obtained by the WT/DSS group, scoring 2 points on Day 4 (Cycle 1), 3 points on Day 26 (Cycle 2), and 1 point on Day 41 (Cycle 3). As for the animals in the KO/DSS group, they scored 1 point only on Day 23 (Cycle 2) and 1 point from Days 38 to 41 (Cycle 3) (*p* < 0.05) compared to KO/Sham (Figure 1C).

The highest scores were obtained by the WT/DSS group, scoring 2 points on Day 4 (Cycle 1) and 3 points on Day 26 (Cycle 2) compared to KO/DSS (*p* < 0.05) (Figure 1C).

### 3.3 Histological Analysis in Experimental Colitis

Using hematoxylin and eosin (HE) staining, we observed the tissue morphology of the distal neck of mice and identified alterations in the experimental groups (WT/SHAM, WT/DSS, KO/SHAM, and KO/DSS).

Figure S2 shows the HE‐stained distal colon of the experimental groups. In the WT/SHAM and KO/SHAM groups, the intestinal layers appeared intact. However, in the WT/DSS and KO/DSS groups, we observed edema mainly affecting the submucosal layer (SM) and the circular muscle layer (CL). This was likely caused by inflammation, which explains the softer stool consistency in the DAI. There was also infiltration of inflammatory cells within the intestinal layers, quite evident in the colitis groups. The mucosal architecture appears to be disrupted, mainly in the crypt region. It is worth noting that none of the analyzed groups showed hemorrhagic foci or tissue destruction of an ulcerative appearance. Table S3 shows these results, in which no significant differences were observed between the WT/SHAM, WT/DSS, KO/SHAM, and KO/DSS groups.

Periodic acid‐Schiff (PAS) staining revealed the presence and morphology of goblet cells in the distal colon of mice from the WT/SHAM, WT/DSS, KO/SHAM, and KO/DSS groups. The section from the WT/SHAM group demonstrates that these cells are regularly present throughout the intestinal epithelium, showing a circular or oval shape, with a normal distribution of goblet cells along the histological section filled with mucus. In the WT/DSS and KO/DSS groups, goblet cells appeared to have smaller quantities and exhibited a more rounded and swollen appearance (Figure S3A–L). Goblet cells in the WT/DSS group showed a significant decrease of 25.6% compared to the WT/SHAM group (*p* < 0.0001). The KO/DSS group showed a significant decrease of 42.3% compared to the KO/SHAM group (*p* < 0.0001). The KO/SHAM group showed a significant decrease of 12.3% compared to the KO/SHAM group (*p* < 0.01) No significant difference was found between the WT/SHAM and KO/SHAM groups (Figure S3M).

Picrosirius staining allowed the observation of collagen fibers in histological sections of the distal colon from the WT/SHAM, WT/DSS, KO/SHAM, and KO/DSS groups. Thick collagen fibers, characterized by red coloration of Type I collagen, were present in the lamina propria and submucosa of all groups. However, the quantity and intensity of these fibers were greater in the WT/DSS and KO/DSS groups. This was mainly evident in the lamina propria and submucosa regions, where there was an increase and disorganization of the lamina propria with an accumulation in the subepithelial region. Under polarized light, thinner fibers with a green coloration typical of Type III collagen were observed in all regions of the intestinal tissue, mainly in the lamina propria and submucosa across all groups. However, a reduction in these fibers is observed in the WT/DSS and KO/DSS groups compared to the animals in the WT/SHAM and KO/SHAM groups (Figure S4).

### 3.4 P2X7R Immunoreactivity Analysis

Immunoreactivity of P2X7R, nNOS, ChAT, β‐Tubulin, and NLRP3 was observed in neurons and GFAP in enteric glial cells of the myenteric plexus. These markers were present in the cytoplasm and plasma membrane of animals from the WT/SHAM, WT/DSS, KO/SHAM, and KO/DSS groups (Figures 2, 3, 4, 5, 6, Figure S5).

**FIGURE 2:**
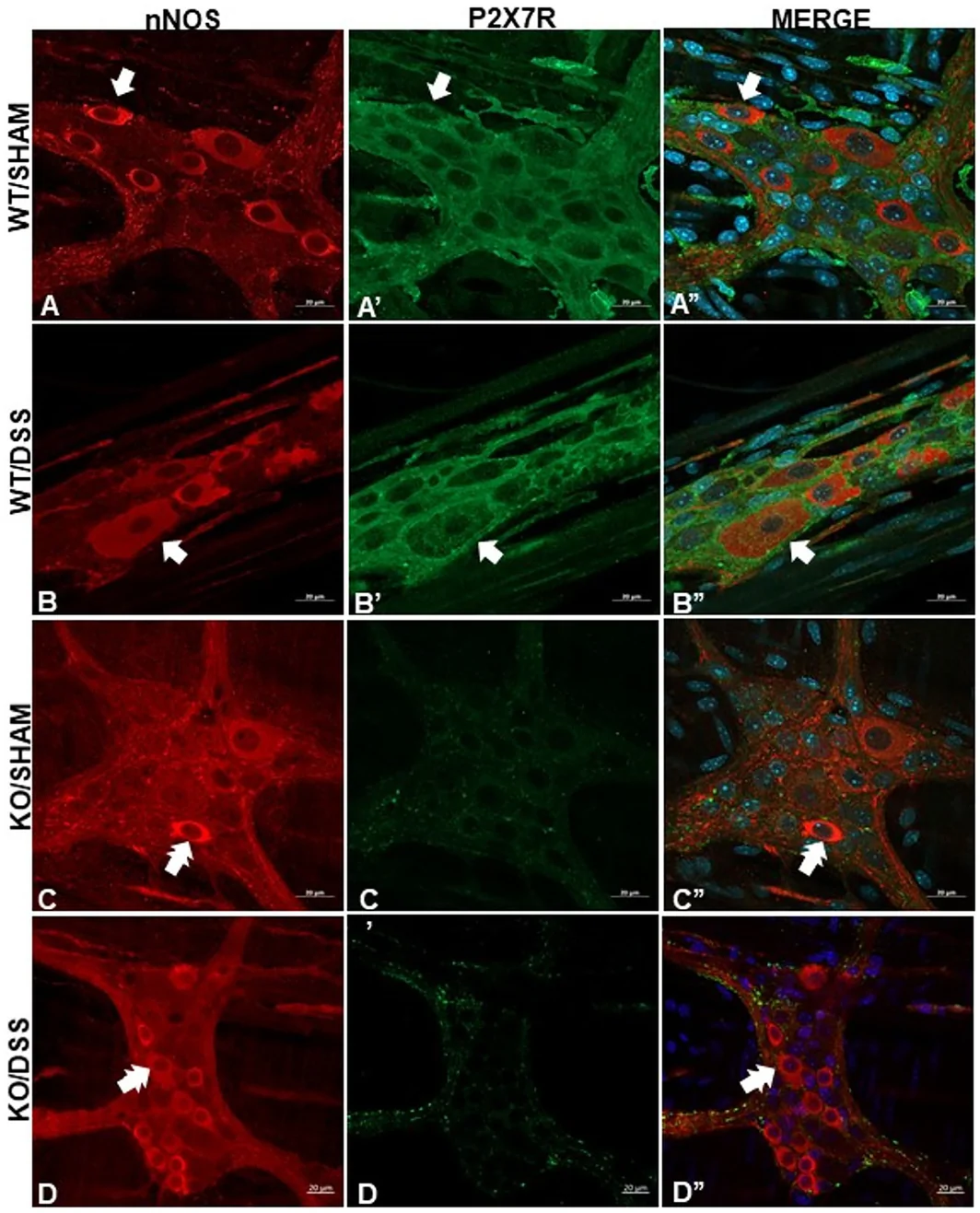
Double labeling of the neuronal nitric oxide synthase (nNOS) with P2X7 receptor (P2X7R) in neurons of the distal colon myenteric plexuses in the WT/SHAM (A, A′, A″) and WT/DSS (B, B′, B″) groups. Colocalization of nNOS (red) and the P2X7 receptor (green). (A″, B″) DAPI (blue) staining for nuclei. Staining of nNOS (red; C, D) in KO/SHAM and KO/DSS mice. (C′, D′) Lack of staining of P2X7 receptor in the KO/SHAM and KO/DSS groups. (C″, D″) DAPI (blue) staining of nuclei. The single arrows indicate double‐labeled neurons staining for nNOS and for the P2X7 receptor. The double arrows indicate labeled neurons only for nNOS (Scale bars = 50 μm).

There was localization of P2X7R with nNOS‐ir neurons in the WT/SHAM and WT/DSS groups (Figure 2). The staining was predominantly in the neuronal cytoplasm. There was no colocalization in the KO animals for P2X7R (KO/SHAM and KO/DSS) due to the absence of P2X7R.

Similar to nNOS‐ir neurons, localization of P2X7R with ChAT‐ir neurons was also observed only in the WT groups (Figure 3). In the case of ChAT neuron labeling, the labeling was predominantly evident in the plasma membrane.

**FIGURE 3:**
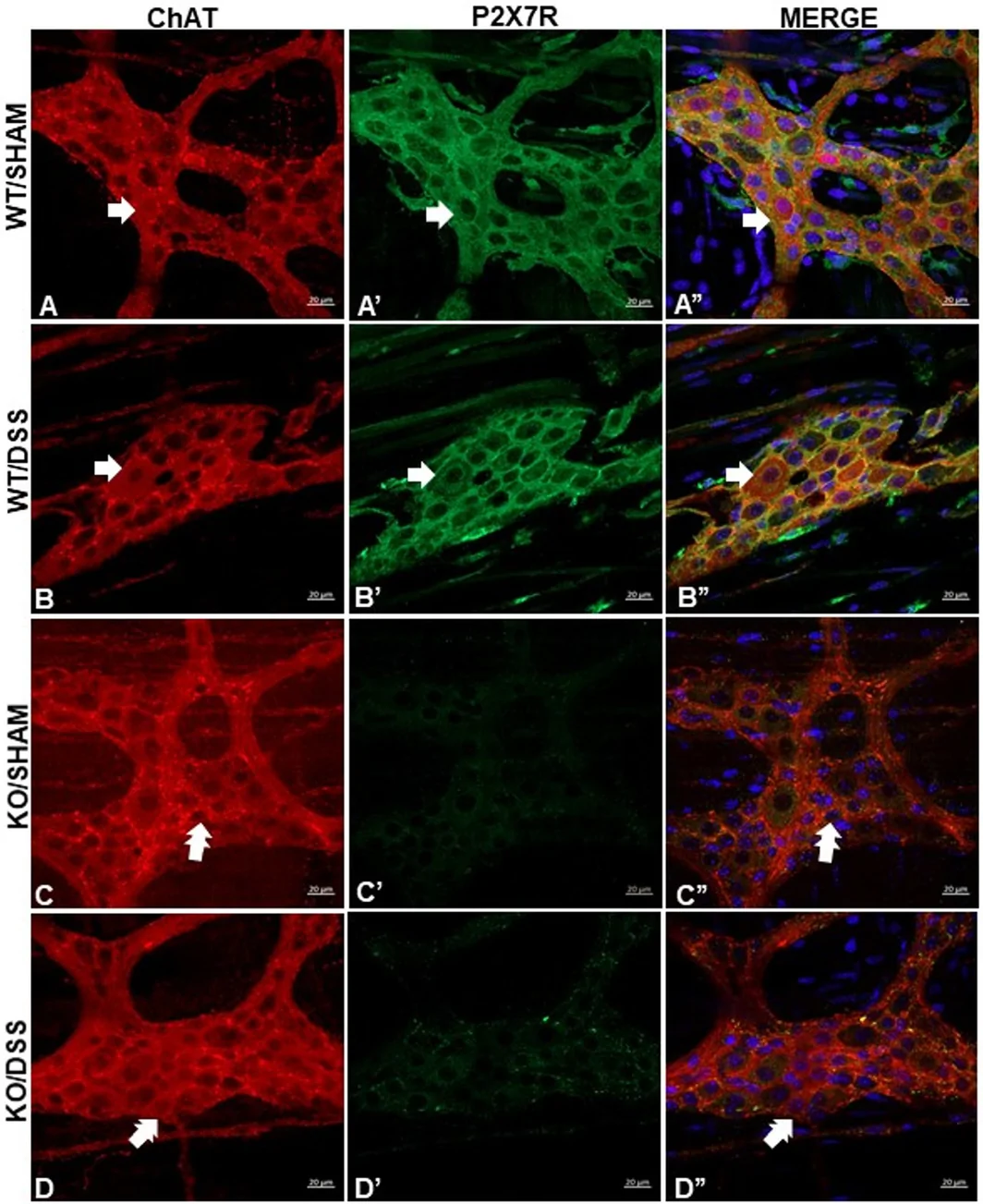
Double labeling of the choline acetyltransferase (ChAT) with P2X7 receptor (P2X7R) in neurons of the distal colon myenteric plexuses in the WT/SHAM (A, A′, A″) and WT/DSS (B, B′, B″) groups. Colocalization of ChAT (red) and the P2X7 receptor (green). (A″, B″) DAPI (blue) staining for nuclei. ChAT (red; C, D) in KO/SHAM and KO/DSS mice. (C′, D′) Lack of staining of P2X7 receptor in the KO/SHAM and KO/DSS groups. (C″, D″) DAPI (blue) staining of nuclei. The single arrows indicate double‐labeled neurons staining for ChAT and for the P2X7 receptor. The double arrows indicate labeled neurons only for ChAT (Scale bars = 50 μm).

Pan‐neuronal β‐Tubulin labeling in the enteric ganglion was evident in all groups studied. The labeling was predominantly in the cytoplasm, and there was no difference in staining between the groups studied (Figure S5). There was no localization of β‐Tubulin neurons with GFAP‐ir enteric glia.

The labeling of enteric glial cells with GFAP was evident in all experimental groups; however, its localization with P2X7R was only possible in the WT groups (Figure 4). GFAP labeling is predominant in the cytoplasm and plasma membrane of glial cells, but this labeling also allows visualization of glial filaments, where glial cells, as one of their characteristics in the face of injury, increase the “arborization” of their filaments as a protective measure [44].

**FIGURE 4:**
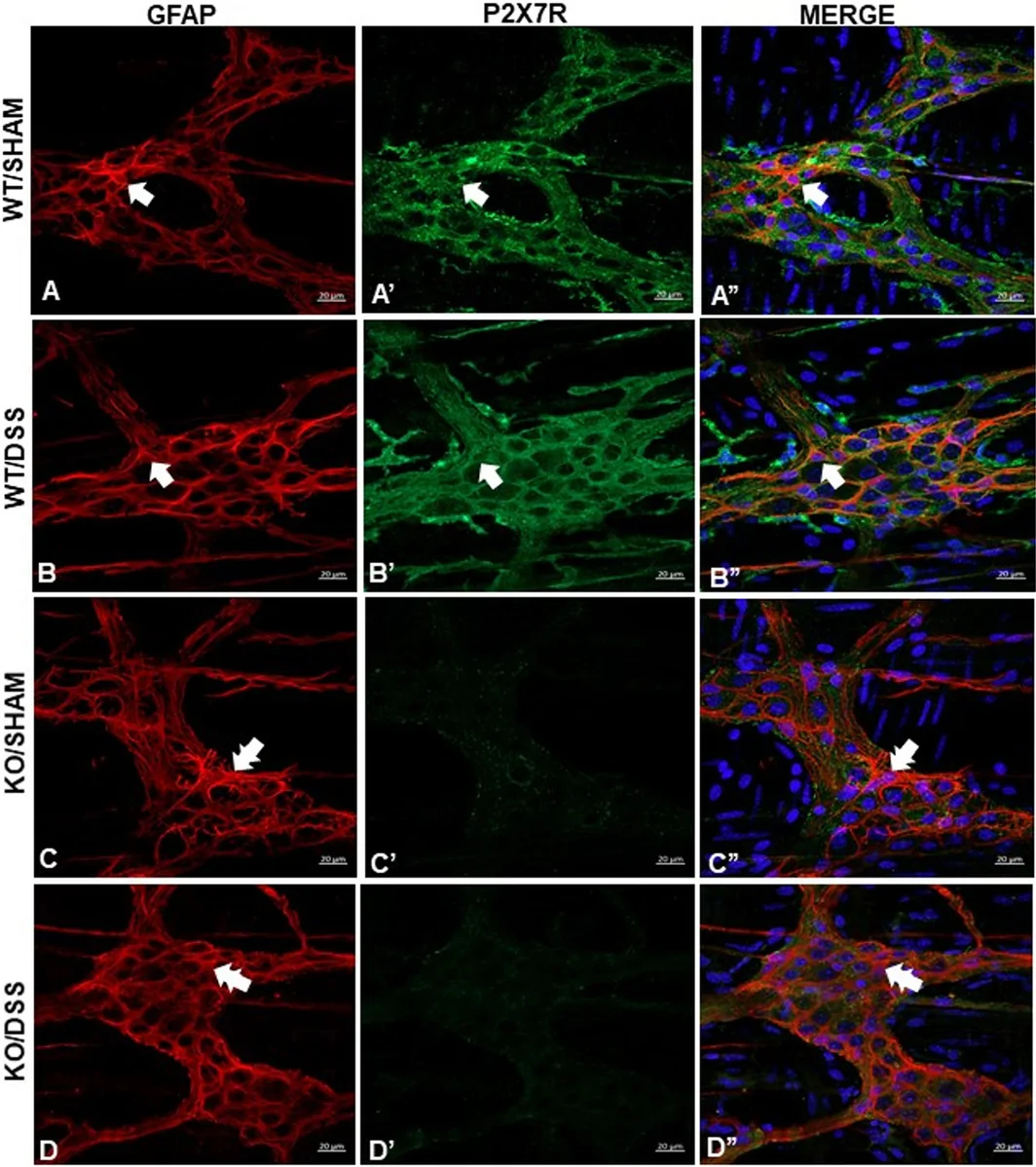
Double labeling of the glial fibrillary protein (GFAP) with the P2X7R of the distal colon myenteric plexuses in the WT/SHAM (A, A′, A″) and WT/DSS (B, B′, B″) groups. Colocalization of GFAP (red) and P2X7R (green). (A″, B″) DAPI (blue) staining for nuclei. GFAP (red; C, D) in KO/SHAM and KO/DSS mice. (C′, D′) Lack of staining of P2X7 receptor in the KO/SHAM and KO/DSS groups. (C″, D″) DAPI (blue) staining of nuclei. The single arrows indicate double‐labeled glial cells staining for GFAP and P2X7R. The double arrows indicate labeled neurons only for GFAP (Scale bars = 50 μm).

The double labeling of GFAP/S100β is shown in Figure S6. In this analysis, enteric glia that were immunoreactive for both GFAP and S100β, for GFAP alone, or for S100β alone were observed. Enteric glia that were immunoreactive for GFAP showed filamentous labeling patterns with many branches (Figure S6).

The proportion of neurons NOSn‐ir neurons that are P2X7 receptor‐positive is 100% in the WT groups. The proportion of ChAT‐ir neurons that is positive for the P2X7 receptor is 100% in WT groups. GFAP‐ir glial cells are 100% positive for the P2X7 receptor in WT groups.

NLRP3 localization was performed with nNOS‐ir and ChAT‐ir neurons for the study of qualitative inflammasome colocalization in neuronal classes of the myenteric plexus in all experimental groups (Figures 5 and 6). NLRP3 labeling is predominant in the plasma membrane and cytoplasm of both neuronal classes (nNOS and ChAT).

**FIGURE 5:**
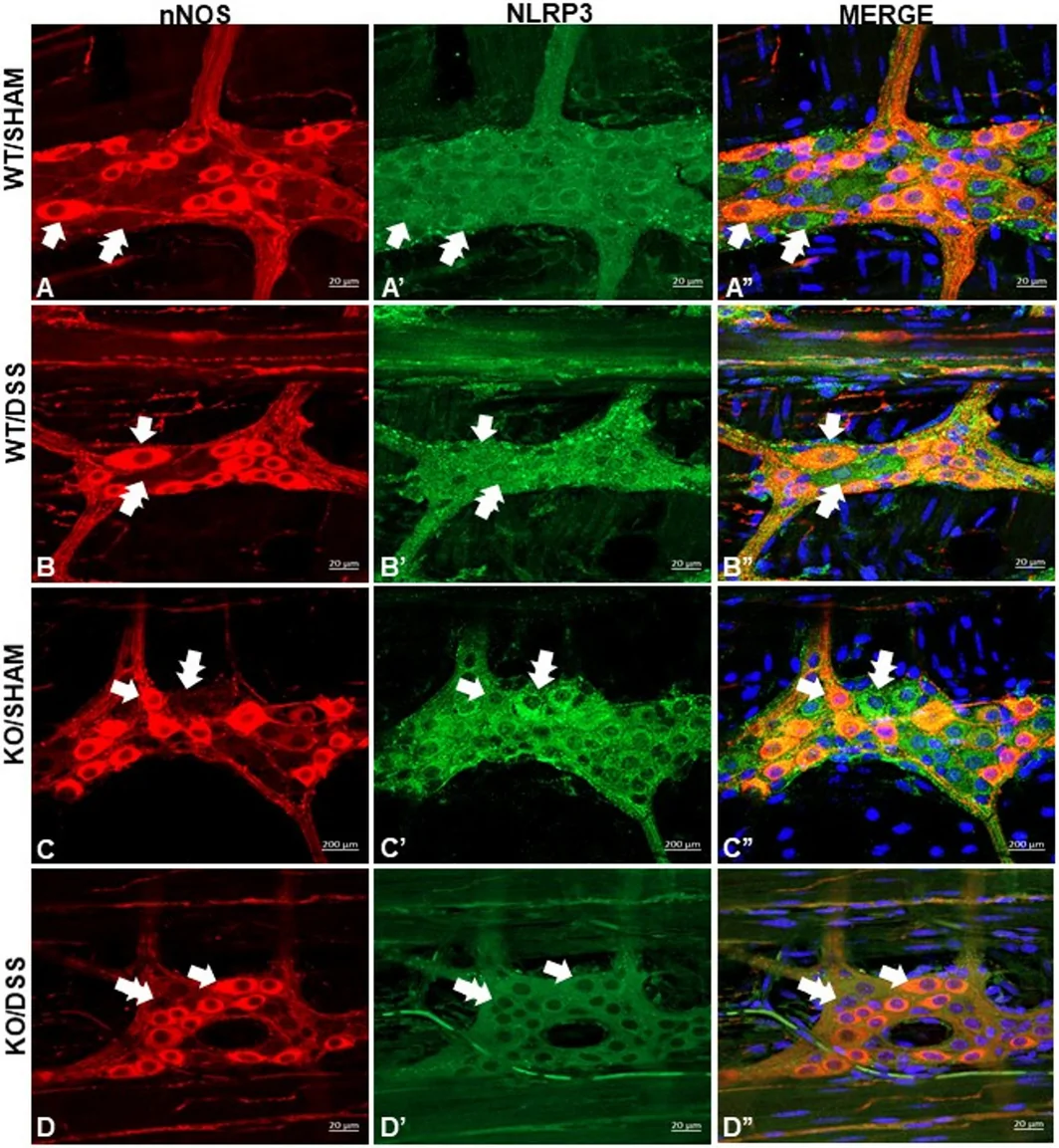
Double labeling of neuronal nitric oxide synthase (nNOS) with the NLRP3 inflammasome of the distal colon myenteric plexuses in the WT/SHAM (A, A′, A″), WT/DSS (B, B′, B″), KO/SHAM (C, C′, C″), and KO/DSS groups (D, D′, D″). Colocalization of nNOS (red) and NLRP3 (green) (A″, B″, C″, D″) DAPI (blue) staining for nuclei. The single arrows indicate double‐labeled neurons staining for nNOS and for NLRP3. The double arrows indicate labeled cells only for NLRP3 (Scale bars = 50 μm).

**FIGURE 6:**
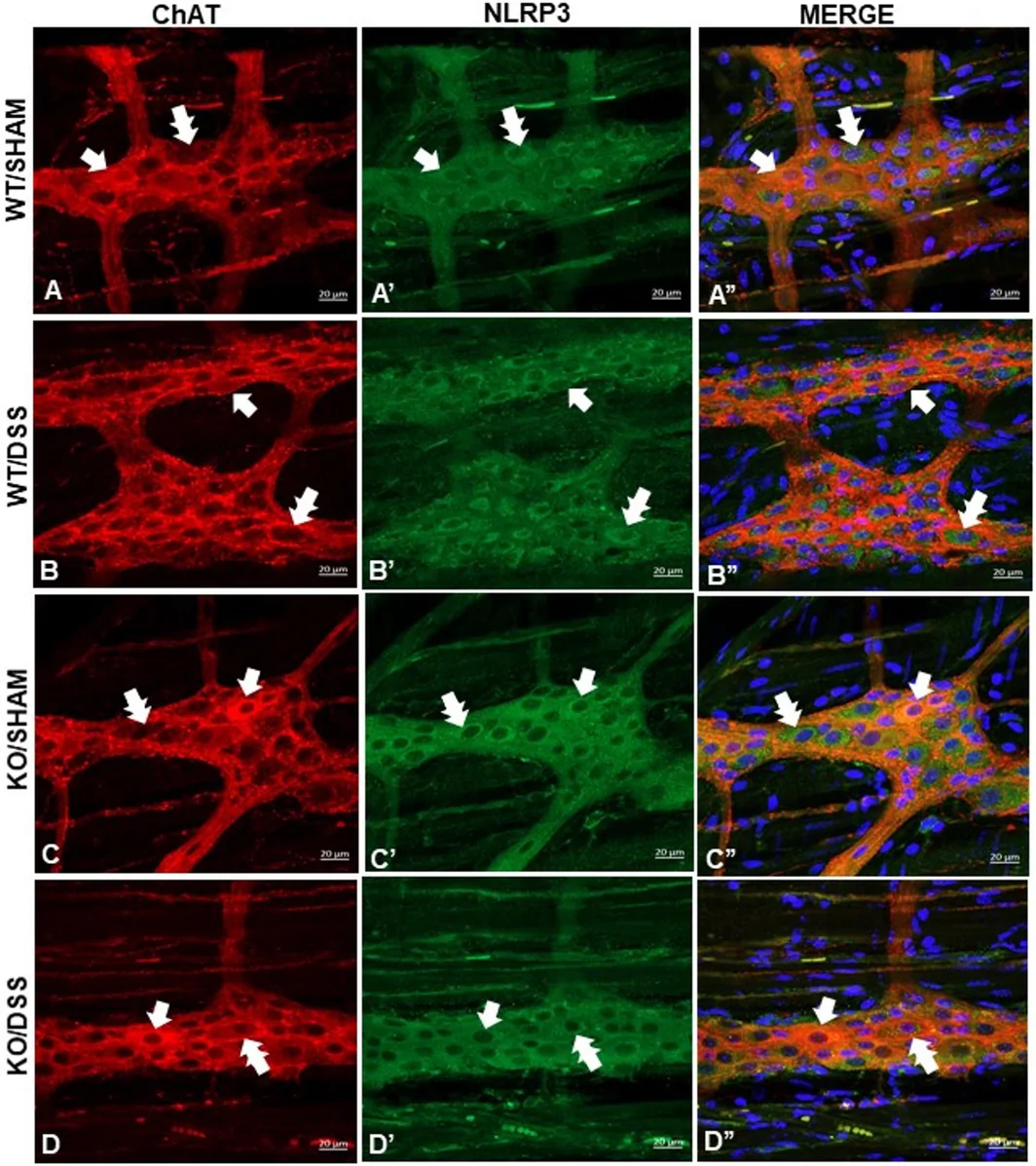
Double labeling of choline acetyltransferase (ChAT) with the NLRP3 inflammasome of the distal colon myenteric plexuses in the WT/SHAM (A, A′, A″), WT/DSS (B, B′, B″), KO/SHAM (C, C′, C″), and KO/DSS groups (D, D′, D″). Colocalization of ChAT (red) and NLRP3 (green) (A″, B″, C″, D″) DAPI (blue) staining for nuclei. The single arrows indicate double‐labeled neurons staining for ChAT and for NLRP3. The double arrows indicate labeled cells only for NLRP3 (Scale bars = 50 μm).

### 3.5 Neuronal Density Analysis

The nNOS‐ir neurons in the WT/DSS group showed a significant decrease of 43.3% compared to the WT/SHAM group (*p* < 0.0001). The KO/DSS group also showed a significant decrease of 32.6% compared to the KO/SHAM group (*p* < 0.0001). There was no significant difference between the WT/SHAM and KO/SHAM groups (Figure 7A).

**FIGURE 7:**
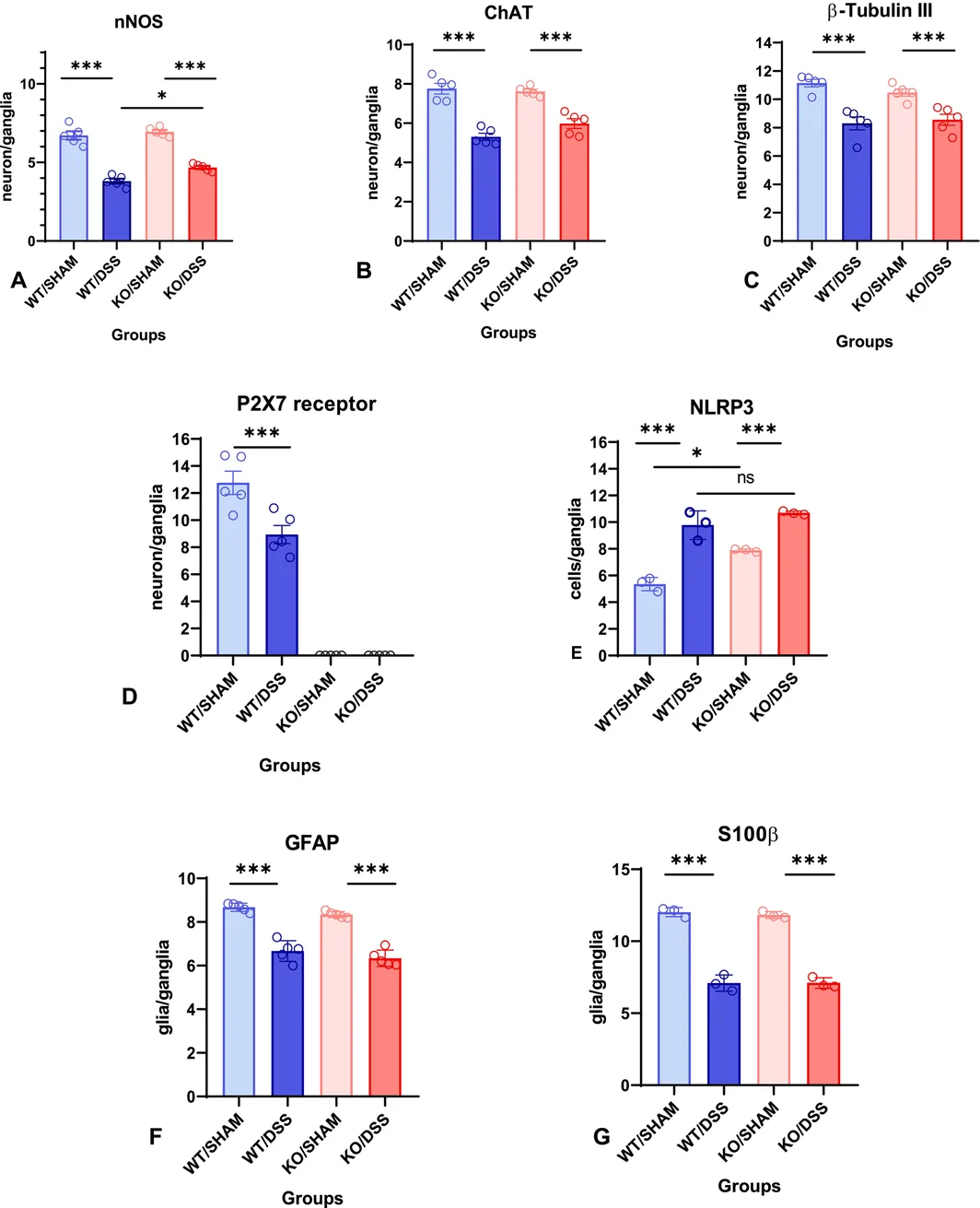
(A–F) Neurons per ganglia of nNOS (A), ChAT (B), pan‐neuronal β‐Tubulin (C), P2X7 receptor (D), NLRP3 inflammasome (E), enteric glial cells GFAP (F), and enteric glial S100β (G) of the myenteric plexus of WT/SHAM, WT/DSS, KO/SHAM, and KO/DSS groups. The data are presented as the means ± SEMs (*n* = 5 or 4 per group) and were compared by two‐way ANOVA and Tukey's multiple comparison test. *WT/DSS versus KO/DSS (*p < 0.01), ***WT/SHAM versus WT/DSS, ***KO/SHAM versus KO/DSS (***p < 0.0001).

ChAT‐ir neurons in the WT/DSS group also showed a significant decrease of 31.6% compared to the WT/SHAM group (*p* < 0.0001). The KO/DSS group showed a significant decrease of 21.6% compared to the KO/SHAM group (*p* < 0.0001). No significant difference was found between the WT/SHAM and KO/SHAM groups (Figure 7B).

The β‐Tubulin–ir neurons in the WT/DSS group showed a significant decrease of 25.4% compared to the WT/SHAM group (*p* < 0.0001). The KO/DSS group also showed a significant decrease of 18.4% compared to the KO/SHAM group (*p* < 0.0001). There was also no significant difference between the WT/SHAM and KO/SHAM groups (Figure 7C).

The P2X7R immunoreactive cells in the WT/DSS group showed a significant decrease of 29.9% compared to the WT/SHAM group (*p* < 0.001). There was no P2X7R staining in the KO/SHAM and KO/DSS groups (Figure 7D).

NLRP3‐ir cells from the WT/SHAM group showed a significant decrease of 45.1% compared to the WT/DSS group (*p* < 0.0001). The KO/SHAM group also showed a significant decrease of 26.2% compared to the KO/DSS group (*p* < 0.0001). NLRP3‐ir cells from the KO/DSS group showed a significant increase of 28% compared to the WT/DSS group (*p* < 0.01).

There was no significant difference between the WT/DSS and KO/DSS groups (Figure 7E).

Enteric glial cells GFAP‐ir from the WT/DSS group showed a significant decrease of 25.4% compared to the WT/SHAM group (*p* < 0.0001). The KO/DSS group showed a significant decrease of 20.1% compared to the KO/SHAM group (*p* < 0.0001) (Figure 7F). Enteric glial cells S100‐ir from the WT/DSS group showed a significant decrease of 40.9% compared to the WT/SHAM group (*p* < 0.0001). The KO/DSS group showed a significant decrease of 39.1% compared to the KO/SHAM group (*p* < 0.0001) (Figure 7G).

The ratio of ChAT‐ir neurons per ganglia to the total number of B‐tubulin neurons was 69.7% ± 2.2% (WT/SHAM group), 64.7% ± 4.2% (WT/DSS group), 72.9% ± 1.4% (KO/SHAM group), and 70.0% ± 1.4% (KO/DSS group) (Figure S7A). The ratio of nNOS‐ir neurons per ganglia to the total number of B‐tubulin neurons was 60.6% ± 3.7% (WT/SHAM group), 46.5% ± 3.3% (WT/DSS group), 66.5% ± 1.9% (KO/SHAM group), and 55.1% ± 2.0% (KO/DSS group) (Figure S7B).

### 3.6 Neuronal Profile Area and Distribution

The nNOS‐ir neurons from the WT/DSS group showed an increase of 35.4% (*p* < 0.0001) compared to the WT/SHAM (Figure 8A). There was no significant difference between the KO/SHAM and KO/DSS groups. ChAT‐ir neurons in the WT/DSS group showed a decrease of 13.2% (*p* < 0.01) and 15.5% (*p* < 0.001) compared to the WT/SHAM and KO/SHAM groups, respectively (Figure 8B). β‐Tubulin neurons in the WT/DSS group showed a decrease of 19.0% (*p* < 0.01) and 16.7% (*p* < 0.01) compared to the WT/SHAM and KO/SHAM groups, respectively (Figure 8C).

**FIGURE 8:**
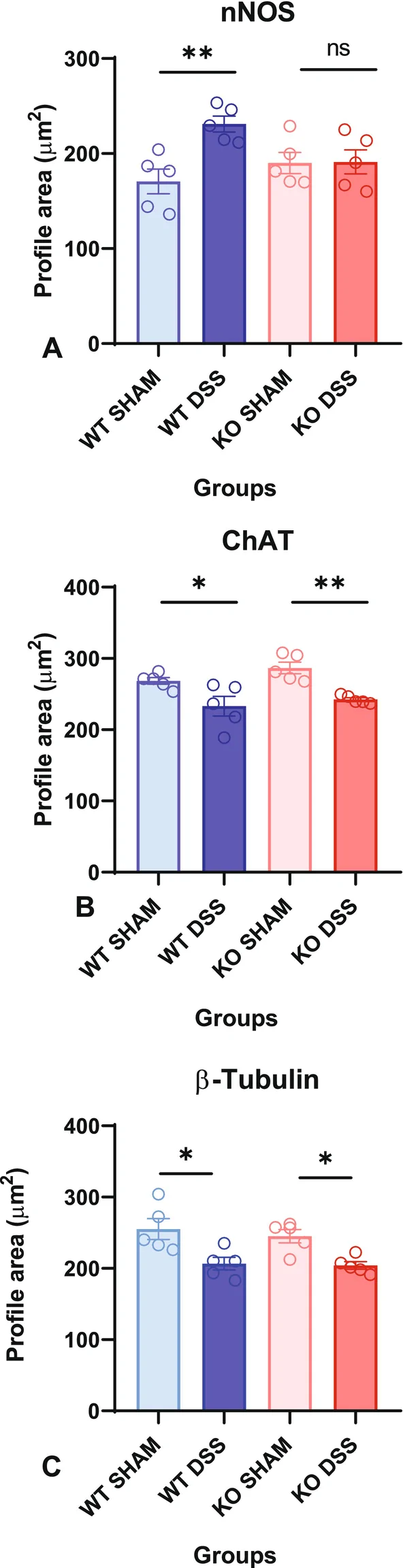
(A–C) Profile area of nNOS (A), ChAT (B), and pan‐neuronal β‐Tubulin (C) of the myenteric plexus of WT/SHAM, WT/DSS, KO/SHAM, and KO/DSS groups. The data are presented as the means ± SEMs (*n* = 4 per group) and were compared by two‐way ANOVA and Tukey's multiple comparison test. *WT/SHAM versus WT/DSS, *KO/SHAM versus KO/DSS (*p < 0.01); **WT/SHAM versus WT/DSS, **KO/SHAM versus KO/DSS (**p < 0.001); ns, no significant difference.

The relative frequency distribution histogram of the areas showed that the largest proportion of nNOS‐ir neurons has an area between 50 and 400 μm^2^. In the WT/SHAM group, a variability of 100–400 μm^2^ was observed, with 70% of the neurons presenting an area between 150 and 200 μm^2^. In the WT/DSS group, the neurons had a variability of 150–400 μm^2^, with 59% of the neurons in the WT/DSS group presenting an area between 200 and 250 μm^2^. In the KO/SHAM group, the variability was from 100 to 350 μm^2^, with 73% of the neurons presenting an area between 150 and 200 μm^2^. Regarding the KO/DSS group, the neurons have a variability of 150–400 μm^2^, with 71% of the neurons in the KO/DSS group having an area between 150 and 200 μm^2^ (Figure S8A).

In the analysis of ChAT‐ir neurons, the area varied between 100 and 550 μm^2^. In the WT/SHAM group, a variability of 150–450 μm^2^ was observed, with 52% of the neurons presenting an area between 200 and 250 μm^2^. In the WT/DSS group, the neurons have a variability between 100 and 450 μm^2^, with 55% of the ChAT‐ir neurons presenting an area between 200 and 250 μm^2^. In the KO/SHAM group, the area variability is between 150 and 550 μm^2^, with 52% of the neurons presenting an area between 250 and 300 μm^2^. Finally, the neurons in the KO/DSS group have an area variability between 150 and 400 μm^2^, with 63% of the neurons having an area between 200 and 250 μm^2^ (Figure S8B).

In the analysis of β‐Tubulin–ir neurons, the area varied between 100 and 400 μm^2^. In the WT/SHAM group, the area varied from 150 to 450 μm^2^, with 55% of the neurons presenting an area between 200 and 250 μm^2^. In the WT/DSS group, the area ranged from 100 to 350 μm^2^, with 63% of the neurons presenting an area between 150 and 200 μm^2^. The KO/SHAM group had an area variability of 150–400 μm^2^, with 65% of the neurons presenting an area between 200 and 250 μm^2^. In the KO/DSS group, the area variability ranges from 100 to 400 μm^2^, with 66% of the neurons showing an area between 150 and 200 μm^2^ (Figure S8C).

## 4 Discussion

Acute or chronic ulcerative colitis induced by DSS is the most widely used murine experimental colitis in IBD research [40, 45]. This model was established by Okayasu in 1990 through the administration of DSS with a molecular weight of 40–50 kDa in drinking water [45, 46].

DSS is a non‐genotoxic pro‐inflammatory agent used to trigger acute and chronic colitis [47]. Administered orally, diluted in drinking water, it causes signs similar to human ulcerative colitis, such as weight loss, bloody diarrhea, loss of epithelial integrity, and accumulation of neutrophilic granulocytes [48]. The compromised barrier integrity following epithelial damage leads to the exposure of mucosal and submucosal immune cells to luminal antigens, causing chronic inflammation after DSS discontinuation [38, 49]. The originality of this manuscript lies in the study of enteric neuronal classes and enteric glia using a DSS‐induced chronic colitis protocol in P2X7 receptor‐deficient mice.

It is known that the severity of experimental disease induction is determined by molecular weight, DSS concentration, and duration of application [40, 48, 50]. In this study, experimental colitis was induced at a dose of 2% in Cycles 1 and 2 and 1.5% in Cycle 03; however, alternating cycles of DSS and water characterized remitting‐recurrent IBD. The presence of loose stools, fecal blood, and weight loss from the fourth day observed in the DAI corroborates the literature on chronic models of ulcerative colitis [51] that used the same concentrations of DSS administration [37, 38, 39].

Experimental studies have demonstrated the role of the P2X7R in the pathophysiology of IBD. In models of experimental ulcerative colitis induced by TNBS (trinitrobenzenesulfonic acid), an increase in the P2X7R was observed in intestinal epithelial cells, which is associated with the release of extracellular ATP in tissue damage and inflammatory stress. Receptor activation contributes to the inflammatory response, in addition to inducing apoptosis and compromising immune regulation. With P2X7 antagonists, the inflammatory response is reduced, demonstrating their activity in pathogenesis and a possible therapeutic target in IBD [20, 30].

The macroscopic changes observed were related to hyperemia and shortening in the intestines of the colitis groups (WTT/DSS and KO/DSS); therefore, in the 3% DSS model, intestinal shortening was observed, but without apparent damage, lesions, or hyperemia [48], which may have been caused by differences in frequency and doses administered.

In the qualitative analysis, colocalization of nNOS and ChAT with the NLRP3 inflammasome was observed, as well as an increase in cells expressing NLRP3 in the WT/DSS and KO/DSS groups, suggesting that the aggressiveness of inflammation caused by chronic experimental ulcerative colitis leads to the activation of other cells, such as enteric glia, by pro‐inflammatory cytokines and interleukins, such as TNF‐α and IL‐1β [52]. The neuronal classes (nNOS, ChAT, and β‐tubulin) showed a reduction in the number of ganglia and neurons in the WT/DSS and KO/DSS groups caused by intestinal inflammation. Other studies in IBD models have found a decrease in the number of ganglia and neurons even in the early stages of IBD, which occurs due to intestinal barrier dysfunction [28, 42, 48, 53], and also of its function [54]. These dysfunctions may persist even after remission of the inflammatory process, given the hyperexcitability of enteric neurons, which leads to impaired intestinal motility [26]. Also, it was observed that the immunoreactivity of P2X7R was in the nNOS‐ir, ChAT‐ir neurons, and glial cells. The Jooss et al. [55] have demonstrated the presence of P2X7 receptors in macrophages and glial cells. In addition, Jooss et al. [56] observed the presence of the P2X7 receptor in whole‐mount myenteric plexus preparations from P2X7‐EGFP BAC transgenic mice.

Ulcerative colitis causes an increase in the influx of immune cells at the onset of the disease, leading to an increase in NO due to increased expression of inducible nitric oxide synthase (iNOS), which is considered cytotoxic to enteric neurons [57]. A decrease in ChAT expression is also observed in IBD, leading to a decrease in ACh (acetylcholine), one of the main neurotransmitters in the central and peripheral nervous system, which is synthesized by cholinergic neurons (ChAT) [58].

In the WT/DSS group, a reduction in P2X7R‐positive cells and enteric glial cells was observed, demonstrating a relationship between purinergic genes and inflammatory genes in enteric glial cells and demonstrating the therapeutic role of P2X7R blockade [59, 60]. Due to recurrent chronic inflammation, it was possible to observe a decrease in enteric neurons that colocalized with P2X7R, and P2X7R deficiency promoted neuronal protection (density graphs of the KO/DSS group) in animals in the KO/DSS group.

The aggressiveness of chronic inflammation induced by DSS led to a decrease in enteric glia. In the present study, double labeling for GFAP and S100β was performed, and the counts also showed similar results, with a decrease in S100‐ir glial cells in the WT/DSS and KO/DSS groups. On the other hand, in models of acute colitis induced by DSS, there is an increase in enteric glia [48], which is associated with its neuroprotective and intestinal barrier homeostasis function [44, 58]. However, the decrease may suggest functional failure due to persistent inflammation. In chronic stages, enteric glia may become less responsive, which implies a loss of their neuroprotective and homeostatic function and favors the persistence of inflammation, indicating that chronic inflammation causes progressive impairment of enteric glia [61].

Under homeostatic conditions, the NLRP3 inflammasome acts as a defender against pathogenic microorganisms in the host through pyroptosis [62, 63]. However, in inflammation, hyperactivation of pyroptosis leads to pathological progression [52, 64, 65], which is consistent with an increase in NLRP3‐ir cells in the WT/DSS and KO/DSS groups, although an increase in NLRP3‐ir was observed in the KO/SHAM group, which may be associated with the activation of other inflammasome pathways [52] in P2X7R knockout animals as a compensatory mechanism for the absence of P2X7R.

In addition, an increase in NLRP3 has been observed in macrophages and microglia in P2X7 knockout mice, demonstrating a possible regulatory effect of the P2X7 receptor on NLRP3 activation [34]. Studies suggest that P2X7 receptor activation is a key factor in NLRP3 activation, leading to caspase‐1 cleavage and IL‐1β release [66, 67].

The neuronal profile has already been evaluated in a 7‐day DSS protocol for the induction of acute experimental ulcerative colitis, and it was found that the neuronal classes had their area reduced, and even after treatment using anti‐TNF‐α, there was no recovery [48]. However, due to continuous damage caused by DSS administration, altered and distinct behavior can be observed, which leads to the consideration that the SNE provides complex defensive mechanisms for the control of motor, secretomotor, and vasomotor functions [2].

Studies have demonstrated differences in the effects of acute and chronic colitis on enteric neurons. da Silva Watanabe et al. [68] compared DSS‐treated C57BL/6 mice with acute and chronic colitis; they observed a significant increase in the ChAT/PGP9.5 ratio in the chronic colitis group, as well as a decrease in the number of contractions throughout the colon. In an acute colitis model [69], colitis was induced in female C3H/HEN mice using dinitrobenzene sulfonic acid (DNBS). The time points studied following DNBS administration were 0.5, 1, 1.5, 2, 3, 4, 6, 12, 24, and 120 h. The results demonstrated a rapid and significant loss of HuC/D‐immunoreactive neuronal cell bodies in the myenteric plexus (a 42% reduction compared to controls). No changes in myenteric plexus density were observed upon analysis via PGP 9.5 immunostaining.

The progressive infiltration of mononuclear inflammatory cells, maintained even during periods of water‐only intake, indicates persistent intestinal lesions. ATP regulates immune cell functions through its purinergic P2 receptors under inflammatory conditions; once activated, immune cells, including macrophages and helper T cells, will release ATP abundantly via ATP release channels and express P2X7 receptor [10, 70]. Dendritic cells lack P2X7R‐dependent maturation signals, impairing proper activation, migration/chemotaxis, and cytokine secretion (such as IFN‐γ and IL‐12), and toward inflammatory or tumor sites [71].

A reduction in PAS‐stained goblet cells was also observed in the colitis groups (WT/DSS and KO/DSS), which compromises the mucosal barrier, leading to greater bacterial exposure. These findings are consistent with the literature on active phases of ulcerative colitis [42, 72, 73]. The reduced number of goblet cells in the KO/Sham group compared to WT/Sham could suggest a decrease in mucus production, which may be a factor contributing to intestinal inflammation in the KO/DSS group [74, 75].

The animals in the colitis group showed an increase in Type I collagen fibers and a reduction in Type III fibers, suggesting intestinal fibrosis and structural tissue damage, which leads to chronic inflammation and greater susceptibility to injury. This fibrosis results from interactions between the extracellular matrix and fibroblasts, cytokines, and other factors [76]. These findings demonstrate that repeated cycles of DSS promote inflammatory and structural changes relevant to the pathophysiology of ulcerative colitis.

Regarding the limitations of the present study, the high proportion of nNOS neurons (Figure 7S) was likely due to the failure to label all β‐tubulin III‐positive neurons in the distal colon region. Another limitation was the inability to analyze experimental colitis in female animals.

## 5 Conclusion

Chronic DSS‐induced ulcerative colitis promotes significant damage in the ENS of the distal colon, characterized by loss of nitrergic, cholinergic, and total enteric neurons, enteric glial cells, and activation of the NLRP3 inflammasome. These findings demonstrate that, in the KO/DSS group, the neuronal loss and DAI scores were less pronounced compared to the WT/DSS group, indicating relative protection. It is worth noting that, due to the severity of the chronic colitis, a certain degree of protection for nNOS neurons was observed in this protocol using P2X7RKO mice. The P2X7R‐mediated pathways may be a promising therapeutic strategy to attenuate IBD. These findings highlight the role of purinergic P2X7R signaling in enteric neuroinflammatory processes associated with ulcerative colitis.

## Author Contributions

Roberta Figueiroa Souza conducted the literature review and analysis and wrote the manuscript; Roberta Figueiroa Souza, Marcos Antônio Ferreira Caetano, Caroline Bures De Paulo, and Patricia Castelucci contributed to data acquisition, analysis, and interpretation. Patricia Castelucci also designed the study, assisted in data acquisition, analysis, and interpretation, performed critical revisions, and reviewed the manuscript for intellectual content. All authors have read and approved the final version of the manuscript for publication.

## Funding

This study was supported by São Paulo Research Foundation (FAPESP, Brazil, 2022/00086‐1, 2022/14406‐8, and 2023/03973‐1), the Coordination for the Improvement of Higher Education Personnel (CAPES, Brazil, 88887.841815/2023‐00), and the National Council for Scientific and Technological Development (CNPq, Brazil, 314993/2023‐1).

## Conflicts of Interest

The authors declare no conflicts of interest.

## Supporting information


**Figure S1:** Percent survival graph in the WT/SHAM, WT/DSS, KO/SHAM, and KO/DSS groups.

**Figure S2:** Mice distal colon sections stained with Hematoxylin & Eosin (H&E) of WT/SHAM (A–C), WT/DSS (D–F), KO/SHAM (G–I), and KO/DSS (J–L) groups. CL, circular layer; LL, longitudinal layer; LP, lamina propria; M, mucous; MM, muscularis mucosa; SM, submucosal. Arrows show the myenteric plexus (Bars: A, D, G, J 50 μm; Bars: B–C; E–F; H–I; K–L 100 μm).

**Figure S3:** Mice distal colon sections stained with Periodic Schiff Acid (PAS) of WT/SHAM (A–C), WT/DSS (D–F), KO/SHAM (G–I), and KO/DSS (J–L) groups. CL, circular layer; LL, longitudinal layer; LP, lamina propria; M, mucous; MM, muscularis mucosa; SM, submucosal. White arrows show the myenteric plexus, and black arrows show goblet cells. (Bars: A, D, G, J 50 μm; Bars: B–C; E–F; H–I; K–L 100 μm). Number of goblets cells (M) of WT/SHAM, WT/DSS, KO/SHAM, and KO/DSS groups. The data are presented as the means ± SEMs (*n* = 3 per group) and were compared by two‐way ANOVA and Tukey's multiple comparison test. ***WT/SHAM versus WT/DSS, ***KO/SHAM versus KO/DSS, ***p < 0.0001, WT/SHAM versus KO/SHAM *(*p* < 0.01).

**Figure S4:** Mice distal colon sections stained with Picrosirius of WT/SHAM (A–C), WT/DSS (D–F), KO/SHAM (G–I), and KO/DSS (J–L) groups. CL, circular layer; LL, longitudinal layer; LP, lamina propria; M, mucous; MM, muscularis mucosa; SM, submucosal. Bars: A, D, G, J 50 μm; Bars: B–C; E–F; H–I; K–L 100 μm.

**Figure S5:** Labeling of the glial fibrillary protein (GFAP) with the pan‐neuronal B‐Tubulin of the distal colon myenteric plexuses in the WT/SHAM (A, A′, A″), WT/DSS (B, B′, B″), KO/SHAM (C, C′, C″), and KO/DSS (D, D′, D″) groups. (A″, B″, C″, D″) DAPI (blue) staining for nuclei. The single arrows indicate glial cells labeled for GFAP. The double arrows indicate neurons labeled only for B‐Tubulin (Scale bars = 50 μm).

**Figure S6:** Double labeling of the glial fibrillary protein (GFAP) with the S100β of the distal colon myenteric plexuses in the WT/SHAM (A, A′, A″), WT/DSS (B, B′, B″), KO/SHAM (C, C′, C″), and KO/DSS (D, D′, D″) groups. Colocalization of GFAP (red) and S100β (green). (A″, B″, C″, D″) DAPI (blue) staining for nuclei. The single arrows indicate double‐labeled glial cells staining for GFAP and S100β. The double arrows indicate labeled neurons only for S100β (Scale bars = 50 μm).

**Figure S7:** (A, B) The ratio of (A) ChAT and (B) nNOS neurons per ganglia to the total number of β‐tubulin in the myenteric plexus of the distal colon of mice in the WT/SHAM, WT/DSS, KO/SHAM, and KO/DSS groups. The data are expressed as mean ± SEM.

**Figure S8:** (A–C) Distribution frequency of nNOS (A), ChAT (B), and pan‐neuronal β‐Tubulin (C) of the myenteric plexus of WT/SHAM, WT/DSS, KO/SHAM, and KO/DSS groups.

**Table S1:** Disease Activity Index (DAI).
**Table S2:** Characteristics of the primary and secondary antibodies used.
**Table S3:** Morphometry of the distal colon muscular, submucosa, crypt depth, and crypt width of distal colon of WT/SHAM, WT/DSS, KO/SHAM, and KO/DSS groups.

## Data Availability

All data are available from the corresponding author upon request.
