# Supplementary material for: Enteric Neurons and Glial Cells Are Partially Protected in P2X7 Receptor‐Deficient Mice With Ulcerative Colitis
Source: Neurogastroenterol Motil. 2026 Nov 10;38(11):e70499. doi: 10.1111/nmo.70499 (PMC13749138; doi:10.1111/nmo.70499)
Supplement: Supplementary file 2 [file NMO-38-e70499-s003.docx]

Figure Supplementary 1
