# Supplementary material for: Enteric Neurons and Glial Cells Are Partially Protected in P2X7 Receptor‐Deficient Mice With Ulcerative Colitis
Source: Neurogastroenterol Motil. 2026 Nov 10;38(11):e70499. doi: 10.1111/nmo.70499 (PMC13749138; doi:10.1111/nmo.70499)
Supplement: Supplementary file 4 [file NMO-38-e70499-s006.docx]

**TABLES**

**Supplementary Table 1. Disease Activity Index (DAI)**

| *Score* | *Weight Loss (%)* | *Stool consistency* | *Rectal bleeding* |
| --- | --- | --- | --- |
| 0 | None | Well-formed pellets | No blood |
| 1 | 1-2 | Normal | Blood traces |
| 2 | 2-4 | Loose and semi-formed stools | Mild bleeding |
| 3 | 4-6 | Liquid stools | Obvious bleeding |
| 4 | >6 | Diarrhea | Gross bleeding |

**Supplementary Table 2. Characteristics of the primary and secondary antibodies used.**

| *Antigen* | *Host* | | | *Dilution* | | *Source* | | Source/Lot. | | RRID | |  | |  | |  | |  | |
| --- | --- | --- | --- | --- | --- | --- | --- | --- | --- | --- | --- | --- | --- | --- | --- | --- | --- | --- | --- |
| P2X7R | | Rabbit | 1:200 | | Millipore | | Merck Millipore (AB5246, lot. 2361386) | | AB_91773 | |  | |  | |  | |  | |  |
| nNOS | | Sheep | 1:1000 | | Millipore | | Merck Millipore (AB1529, lot. 2488802) | | AB_90743 | |  | |  | |  | |  | |  |
| ChAT | | Goat | 1:100 | | Millipore | | Merck Millipore (AB144P, lot. 1978747) | | AB_90743 | |  | |  | |  | |  | |  |
| Β-Tubulin III | | Rabbit | 1:100 | | Cell Signaling | | Cell Sig.  (D7169, lot.  5568S) | | AB_10694505 | |  | |  | |  | |  | |  |
| GFAP | | Goat | 1:500 | | Dako | | DAKO (Z0334/lot. 00061481 | | AB_10013382 | |  | |  | |  | |  | |  |
| NLRP3 | | Rabbit | 1:100 | | Sigma-Aldrich | | Sigma-Aldrich  (SAB5700723, lot. 0600560401) | | AB_3663044 | |  | |  | |  | |  | |  |
| *Secondary antibodies* | |  |  | |  | |  | |  | |  | |  | |  | |  | |  |
| Donkey anti-rabbit IgG 488 | |  | 1:500 | | Molecular Probes | | Molecular Probes (A21206, lot. 1182675) | | AB_2535792 | |  | |  | |  | |  | |  |
| Donkey anti-sheep IgG 594 | |  | 1:100 | | Molecular Probes | | Molecular Probes (A11016, lot. 93D1-1) | | AB_2534083 | |  | |  | |  | |  | |  |
| Donkey anti-goat IgG 594 | |  | 1:300 | | Molecular Probes | | Molecular Probes (A11058, lot. 1608643) | | AB_2534105 | |  | |  | |  | |  | |  |
|  | |  |  | |  | |  | |  | |  | |  | |  | |  | |  |

RRID = Research Resource Identification Portal <https://www.rrids.org/>

**Supplementary Table 3.** Morphometry of the distal colon muscular, submucosa, crypt depth and crypt width of distal colon of WT/SHAM, WT/DSS, KO/SHAM, and KO/DSS groups.

| **Parameters (µm)** | **WT/SHAM** | **WT/DSS** | **KO/SHAM** | **KO/DSS** |
| --- | --- | --- | --- | --- |
| **Muscular** | 13.5 ± 0.8 | 15.1 ± 3.3ns | 13.5 ± 1.3 | 17.8 ± 1.7 ns |
| **Submucosa** | 3.5 ± 0.3 | 5.7 ± 0.9 ns | 6.1 ± 0.8 | 5.8 ± 0.3 ns |
| **Crypt depth** | 10.1 ± 0.1 | 11.7 ± 0.6 ns | 11.8 ± 0.8 | 12.3 ± 0.4 ns |
| **Crypt width** | 4.9 ± 0.3 | 6.5 ± 0.5 ns | 5.5 ± 0.1 | 6.6 ± 0.5 ns |

The data are presented as the means ± SEMs (n=3 per group) and were compared by two-way ANOVA and Tukey’s multiple comparison test *(ns =* no significant difference*).*
